# Transforming academic public health mentorship: implementation and expansion of MOSAIC

**DOI:** 10.3389/fpubh.2025.1640606

**Published:** 2025-11-28

**Authors:** Goleen Samari, Daniel Soto, Kelsie Campbell, Evelyn Gonzalez, Ricky N. Bluthenthal, Stephanie A. Grilo

**Affiliations:** 1 Department of Population and Public Health Sciences, Keck School of Medicine, University of Southern California, Los Angeles, CA, United States; 2 Heilbrunn Department of Population and Family Health, Mailman School of Public Health, Columbia University, New York, NY, United States

**Keywords:** mentorship, mentorship program, anti-racism and equity, faculty and student interaction, graduate student mentoring, public health education

## Abstract

Persistent systemic inequities disproportionately impact BIPOC, first-generation, and international students in graduate public health education. Despite institutional diversity, equity, and inclusion commitments, these students frequently encounter structural barriers such as limited mentorship, inadequate faculty representation, marginalization, and restricted access to academic resources. The Mentoring of Students and Igniting Community (MOSAIC) program addresses these inequities through structured faculty-student group mentorship, professional development, and peer support networks. Recognizing mentorship’s vital role in fostering academic success and identity affirmation, MOSAIC employs a socio-ecological approach, systematically targeting barriers across macro, meso, and micro levels to cultivate inclusive academic environments. This manuscript describes the adaptation and implementation of MOSAIC within the Department of Population and Public Health Sciences (DPPHS) at the University of Southern California (USC), emphasizing support for doctoral students. MOSAIC’s USC adaptation integrates institutional commitment, targeted funding, and dedicated faculty leadership, explicitly engaging faculty who share identities with students. Through structured mentorship programming, responsive student-driven workshops, and community-building activities, MOSAIC aims to facilitate career preparedness, demystify the hidden curriculum, and enhance students’ overall well-being and belonging. In its first year, MOSAIC at USC engaged 15 to 20 doctoral students, and strengthened faculty engagement with anti-racist pedagogy and mentorship. The initiative bridged doctoral programs within DPPHS, promoting inter-program collaboration and fostering a supportive, inclusive community. Early feedback from students indicates improved institutional climate perceptions, stronger mentor-student relationships, increased academic self-efficacy, and reduced isolation among participants. The successful USC adaptation demonstrates MOSAIC’s transferability and underscores structured mentorship’s potential for catalyzing broader institutional transformation. The manuscript offers detailed insights and a replicable programmatic framework, guiding institutions seeking to adopt the MOSAIC program. By embedding anti-oppressive strategies and striving for sustained institutional commitment, MOSAIC contributes significantly to reshaping public health education toward equity, inclusivity, and justice-oriented mentorship, ultimately preparing a diverse workforce to address complex public health challenges.

## Introduction

The pursuit of graduate education in public health remains marked by persistent inequities disproportionately affecting Black, Indigenous, and People of Color (BIPOC), first-generation (first-gen), and international students, underrepresented groups that share identities and comparable experiences navigating post-secondary educational environments (1–3). Despite institutional commitments to diversity, equity, and inclusion (DEI), these students frequently encounter structural and systemic barriers that hinder their academic progression and professional growth. Key challenges include inadequate mentorship, limited engagement with faculty of similar backgrounds, difficulty accessing academic resources, and pervasive experiences of marginalization that impact their sense of belonging and overall mental health (1, 4–6). These compounded barriers translate into heightened stress, lower retention rates, and a less positive overall graduate experience for BIPOC students (1, 5). These challenges are compounded for doctoral students, whose academic and professional success heavily relies on robust mentorship relationships and supportive institutional structures (7, 8).

Mentoring or “the process by which a novitiate… is positively socialized by a sagacious person for the purpose of learning the traditions, practices, and frameworks of a profession, association, or organization” is recognized as a crucial aspect of career and professional development (9). Historically excluded and underrepresented students and faculty do not have comparable access to role models, mentors, and informal networks that are critical to academic success and career advancement (10–12). At the same time, it is established that mentorship, particularly research mentorship, plays a role in the formation of scientific identity (13). Mentor and mentee relationships have proven particularly salient for the retention and success of historically underrepresented students as they often lack access to informal professional and academic networks and information needed to navigate institutional environments (14, 15). While a substantial, ongoing conversation exists on mentoring graduate students and junior faculty, the literature on mentoring first generation, BIPOC, and international students, particularly graduate students, is not as robust (1, 5, 12, 15, 16). Yet research has established that BIPOC, first-generation, and international students facing similar barriers have the most to benefit from graduate mentoring programs that are designed to help students navigate predominantly white institutions (PWI) (17).

Recognizing that mentorship is central to addressing educational disparities and experiences (18, 19), innovative pedagogy, curricula, and programs that explicitly embed anti-racist principles and culturally responsive practices have emerged (8, 20, 21). Among these, the Mentoring of Students and Igniting Community (MOSAIC) program, initially developed at Columbia University’s Mailman School of Public Health (MSPH), has shown promise in addressing these systemic inequities through intentional faculty-student mentorship, professional development, and peer-support networks (21). Since its inception, MOSAIC has expanded rapidly, from serving 26 students in 2019 to over 450 participants by 2024, and has been associated with increased graduate school satisfaction, a stronger sense of belonging, and improved quality of life (22). These promising MOSAIC outcomes reflect the broader call within public health education to integrate anti-oppression and anti-racist strategies into teaching, community engagement, and doctoral training (8, 20, 23–25).

The original development of MOSAIC at Columbia was informed both by existing literature on mentorship of graduate students (1, 5, 15, 16, 21, 26, 27) and a broader literature that understands structural racism, sexism, and intersectionality to be multilevel and intersectional in its impacts on health and well-being (28–31). Research suggests that effective mentoring for marginalized students requires an intentional socio-ecological (macro, meso, and micro) approach to create a supportive “mentoring ecosystem” (19, 32, 33) and that holistic faculty support has academic, psychosocial, and sociocultural dimensions (26). Building on these concepts, the MOSAIC program conceptualizes barriers faced by BIPOC, first-generation, and international students across multiple interconnected levels: macro (i.e., policy/societal and institutional), meso (i.e., community and relationships), and micro (i.e., individual).

At the macro-level (policy/societal), structural racism and historic marginalization perpetuate systemic inequities that manifest as oppressive academic institutional systems and institutional power imbalances. These macro factors lay the foundation for institutional barriers, including exclusionary access to the hidden curriculum, limited formal institutional support, and underrepresentation of first-generation students and faculty of color. These structures operate concurrently through routes within meso or community and interpersonal relationship contexts like interpersonal discrimination, lack of peer community, limited informal access to faculty, and daily aggressions. To create micro-level or individual impacts, these multilevel barriers reinforce discriminatory experiences attributable to intersectional identities and experienced through formal and informal mechanisms of racialization. These pathways negatively affect students’ quality of life, health, sense of belonging, career preparedness, and overall academic progress.

**Figure 1:**
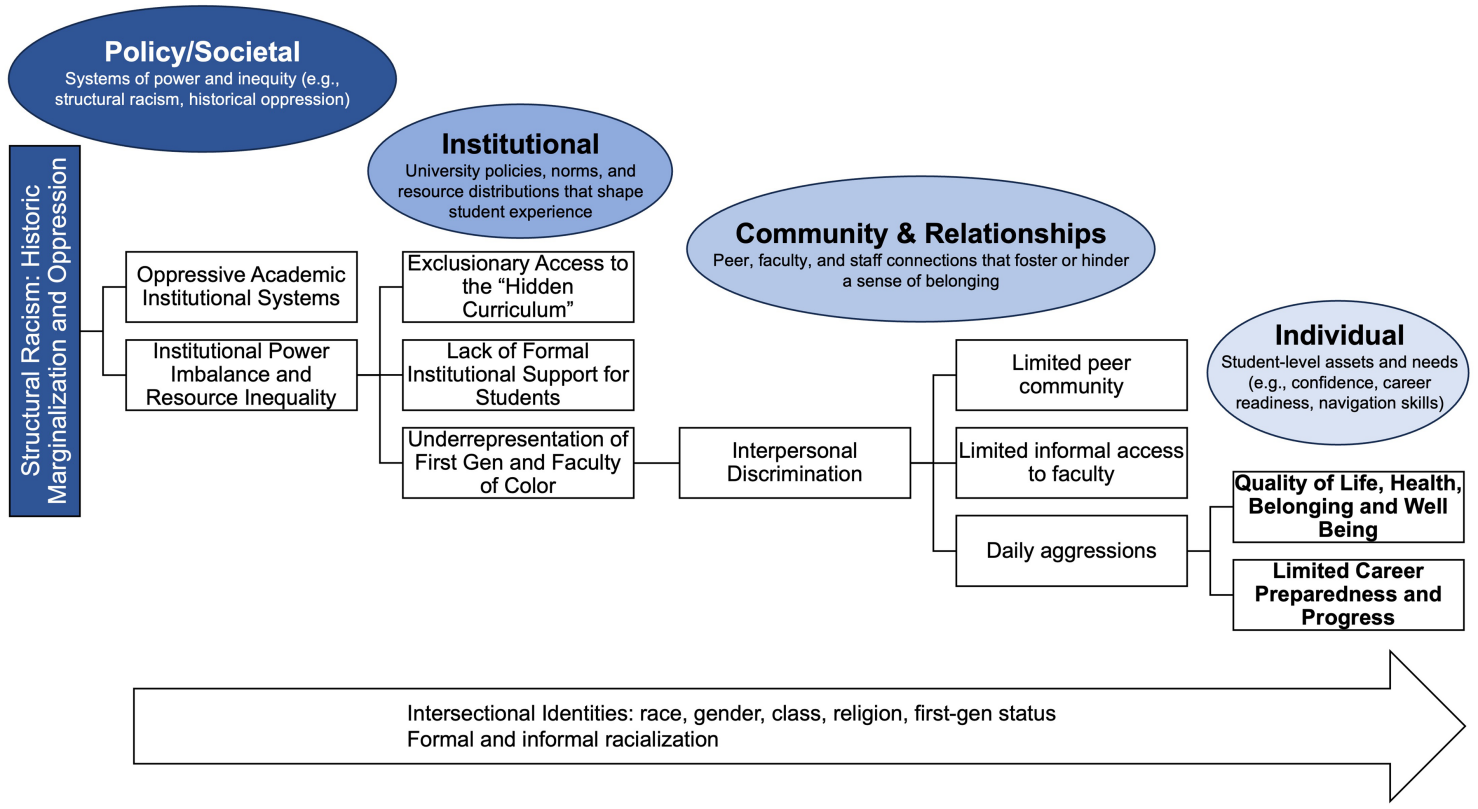
Conceptual model of barriers.

While previous evaluations have highlighted MOSAIC’s effectiveness at Columbia (22), its programmatic framework and adaptability at other institutions remain less understood. Further, despite growing awareness of systemic inequities, many graduate programs lack comprehensive mentoring structures and programs, particularly at the doctoral level (7, 8, 26). In response to this gap and leveraging the multilevel conceptual framework of barriers (Figure 1), MOSAIC was adapted and implemented at the University of Southern California (USC) to specifically meet the unique needs of doctoral students within a different institutional culture and academic environment. USC’s Department of Population and Public Health Sciences (DPPHS) presents different structural challenges and opportunities, necessitating an adapted mentorship framework that accounts for variations in institutional culture, faculty composition, and doctoral student needs. Guided by principles of socio-ecological theory and racial equity, the USC MOSAIC adaptation emphasizes responsive mentoring practices, targeted professional development for doctoral-level students, and alignment with institutional DEI initiatives.

This paper describes the development and implementation of the USC MOSAIC program, providing a detailed programmatic framework that elucidates how structured mentorship can effectively disrupt systemic barriers faced by BIPOC, first-generation, and international doctoral students in public health. By focusing on adaptive program design, institutional integration, and sustained faculty engagement, we demonstrate how one tailored mentorship initiative can aim to foster inclusive academic environments, enhance doctoral student experiences, and cultivate public health professionals and ultimately, careers. The insights and programmatic framework presented here offer a replicable model for other institutions aiming to implement and adapt MOSAIC to provide robust mentorship, inclusive excellence, and belonging for graduate public health students.

## Methods

MOSAIC’s expansion at USC followed an adaptive change strategy designed to address the mentorship gaps for BIPOC, first-generation, and international doctoral students. The co-directors of MOSAIC at USC conducted a needs assessment through meetings with department administration to identify challenges and opportunities for improvement in mentorship structures and integration into existing diversity, equity, and inclusion (DEI) efforts. Based on these meetings, the initial year of MOSAIC was to focus on doctoral students. USC’s Department of Population and Public Health Sciences has 116 doctoral students across three doctoral programs (Biostatistics, Epidemiology, and Health Behavior Research). Across all three programs, there are 85 doctoral students who identify as BIPOC, 27 who identify as white, and 4 whose race/ethnicity is unknown. The focus on doctoral students at USC was a pivot from the Columbia MSPH MOSAIC, which focuses on masters-level students.

To start to ensure long-term sustainability, the program was aligned with USC’s broader DEI initiatives, securing institutional support and integration into the Department of Population and Public Health Science’s Representation, Engagement, Development, and Impact (REDI) Council. Based on an understanding of the MOSAIC program at Columbia MSPH and to inform the implementation and adaptation of MOSAIC among USC DPPHS doctoral students, a programmatic model for MOSAIC was developed by the co-directors of MOSAIC at USC and Columbia. The programmatic model was informed by an understanding of the multilevel barriers faced by BIPOC, first-generation, and international graduate students in institutions of higher education (Figure 1). Lastly, as standard practice for MOSAIC, USC MOSAIC developed a structured, yet flexible curriculum based on student input that provides targeted support in research mentorship, career preparation, and mental health, tailored to the unique needs of doctoral students.

## Results

### The MOSAIC program model

The MOSAIC Program Model illustrates the multi-level design and implementation strategy of MOSAIC, Mentoring of Students and Igniting Community, a group mentorship program for faculty-to-student mentorship for BIPOC, first-gen, and international graduate students. The MOSAIC Program Model is informed at multiple levels by the conceptual framework of barriers for graduate public health students (Figure 1). The MOSAIC program framework integrates institutional, community and relationship, and individual components to address multi-level barriers comprehensively. The MOSAIC program framework is designed for public health graduate students and the student population served depends on the implementing institution (e.g., Columbia uses this model to implement MOSAIC for master’s students and USC uses this model to implement MOSAIC for doctoral students).

At its foundation, the model begins with an institutional commitment to equity, represented by two primary structural supports: an institutional champion embedded in administration and dedicated financial resources. Institutional champions can be Department Chairs, Vice or Associate Deans, etc., but it is somebody who has access to leadership and resource discussions at the department or school of public health level. These foundational elements enable the appointment of faculty leadership, including co-directors who are faculty of color and/or first-generation scholars, as well as a broader base of faculty mentors drawn from across academic programs and departments. Recognizing the “cultural tax” inherent in many mentorship models, particularly for diverse faculty members who share similar identities with BIPOC, first-generation, and international students, MOSAIC prioritizes stipends and recognition for faculty mentors. Ultimately, this approach benefits faculty, students, mentorship outcomes and signals institutional commitment to inclusive learning environments for underrepresented students. Together, these structural supports and personnel establish the organizational infrastructure for the program’s activities.

Building on this foundation, the program implements a series of student-centered and equity-oriented activities. These include administrative and logistical support, responsive student programming, asynchronous access to resources, research and evaluation components, informal peer engagement opportunities, and intentional community-building between faculty and students. These activities aim to promote organization and structured programming, student agency and engagement, access to academic and research tools, transparency in academic expectations, and social support. Each cluster of activities contributes in complementary ways to fostering a climate of academic belonging and equitable opportunity for BIPOC, first-generation, and international graduate students.

The outcomes of these programmatic activities are grouped into three overarching impact domains: career preparedness, demystification of the hidden curriculum, and enhanced quality of life, belonging, and well-being. These domains reflect the ultimate goals of the MOSAIC program to not only support individual student success but also to catalyze cultural change within the institution. The framework makes clear that these outcomes are a product of layered and reinforcing structures, starting from institutional support and faculty commitment, channeled through targeted program activities, and culminating in long-term developmental, academic, and psychosocial benefits for students (Table 1).

**Table 1:** MOSAIC inputs and outputs.

Level	Barrier	MOSAIC activities	Outputs	Impacts
Macro Policy/Societal	Structural racism and historic resource deprivation perpetuate systemic inequities for BIPOC students	Anti-racist institutional commitment	Institutional buy-in	Diversified academia and workforce
	Pipeline drop-off and lack of BIPOC, first-gen, and international doctoral participation	Multi-stakeholder engagement	Cross-institutional interest	Shift in culture
		Dissemination and advocacy for policy change	Policy changes	Broader health equity impacts
Institutional	Limited institutional support and a PWI campus culture	Structured mentorship programming Group & one-on-one mentoring Formalized department or program mentor Accountability protocols & incident management	Inclusive climate perception	Higher retention and completion
	Exclusionary access to the “hidden curriculum” for BIPOC, international and first-generation students	Academic support infrastructure (CRREEE-aligned resources)	Resource connectivity	Accountability and pipeline growth
	Lack of formal mentoring structures and few identity-affirming faculty role models	Faculty training and involvement	Enhanced faculty-student engagement	Institutional change
Meso (Community/Relationships)	Difficulty building meaningful mentoring relationships with faculty	Faculty-student mentoring activities	Stronger mentoring relationships	Lasting mentoring networks
	Lack of community and identity-affirming peer support	Peer cohort bonding	Sense of belonging and peer support	Career advancement and collaboration
	Limited access to academic social networks	CRREEE student-input sessions; community-building & safe-space events	Identity affirmation	Reduced social capital gap
		Alumni and guest speaker engagements	Improved interpersonal skills and confidence	
Micro individual	Imposter syndrome, stress, and psychosocial needs	Professional and research development	Increased self-efficacy and confidence	Academic achievement and completion
	Limited exposure to the “hidden curriculum”	One-on-one academic guidance	Identity affirmation and reduced isolation	Career readiness and success
	Discrimination-related mental-health burdens (higher anxiety, depression)	Identity and wellness support	Improved academic satisfaction and well-being	Empowerment to mentor others
	Financial pressures & family responsibilities	Material/financial support information	Skill and knowledge gains	

At the broadest structural level, MOSAIC targets pervasive structural racism and inequitable resource distribution that create systemic inequities for BIPOC and international students. To address this, MOSAIC engages in anti-racist institutional commitment, aligning with department or institution-wide initiatives (such as USC’s REDI Council) and embedding equity principles in policies and culture. Additionally, MOSAIC emphasizes multi-stakeholder engagement, involving faculty, staff, and alumni in mentorship programming and advocacy for BIPOC, first-generation, and international students. Through active dissemination and advocacy efforts, MOSAIC seeks policy changes to enhance resources, institutional buy-in, cross-institutional interests, and a broader impact on public health leadership and representation.

At the institutional level, MOSAIC addresses barriers including limited institutional support, PWI campus cultures, and implicit “hidden curricula” or the assumed norms and often unspoken lessons of academia, which particularly disadvantage BIPOC, first-generation, and international doctoral students. This is compounded by a lack of formal mentorship systems, few same-race/first-generation faculty role models, and limited opportunity for peer-to-peer community mentorship. MOSAIC implements structured mentorship programming with group mentorship, formalized department and/or program mentor matching, individual and collective academic guidance, and clear protocols for accountability and incident management. This structured support results in a more inclusive climate perception, stronger relationships between faculty and students, and enhanced institutional commitment to addressing structural inequities.

Interpersonally, BIPOC, international, and/or first-generation students often struggle to establish meaningful mentoring relationships, lack a sense of community, and experience cultural disconnects that strain faculty interactions, which are frequently exacerbated by daily aggressions. This leads to isolation and limited access to academic social networks. MOSAIC addresses these difficulties through faculty-student mentorship activities with BIPOC and first-generation faculty, regular networking events with MOSAIC faculty and peers, community-building and safe-space interactions, as well as alumni and guest speaker engagements. Through these events, MOSAIC strengthens personal and professional connections. MOSAIC aims to equip faculty members with the skills and tools necessary to mitigate the institutional barriers faced by BIPOC, first-generation, and international mentees, including race- and identity -based bias and discrimination, imposter syndrome due to “hidden curricula,” and the absence of inclusive institutional culture and services. Immediate outcomes include deeper, trusting mentor-student relationships, affirmation of identity, and improved interpersonal skills. Over time, these activities can lead to lasting mentorship networks, sustained career support after graduation, reduced social capital gaps, and enhanced career advancement opportunities through collaborative networks and advocacy for equity in academia.

Finally, at the individual level, MOSAIC confronts barriers related to psychosocial challenges, hidden academic curricula, mental health struggles due to discrimination, and financial pressures disproportionately experienced by BIPOC, first-generation, and international doctoral students. MOSAIC provides professional and research development opportunities, explicit one-on-one academic guidance, safe-space discussions, and celebrations of identity affirmation. MOSAIC can also offer tangible material and financial support through scholarships, paid positions, and funded travel when budgets allow. These interventions increase student self-efficacy, confidence, and satisfaction, improving mental health and academic performance. Immediate outcomes include increased self-efficacy and confidence in academic skills, profound identity affirmation and reduced isolation, and improved academic satisfaction and well-being. In the long term, students are more likely to achieve timely academic milestones, complete their programs, and successfully enter the public health workforce. This empowerment model fosters a cycle in which alumni are more likely to mentor junior colleagues, thereby multiplying MOSAIC’s anti-racist impact across the field.

### MOSAIC adaptation at USC

**Figure 2:**
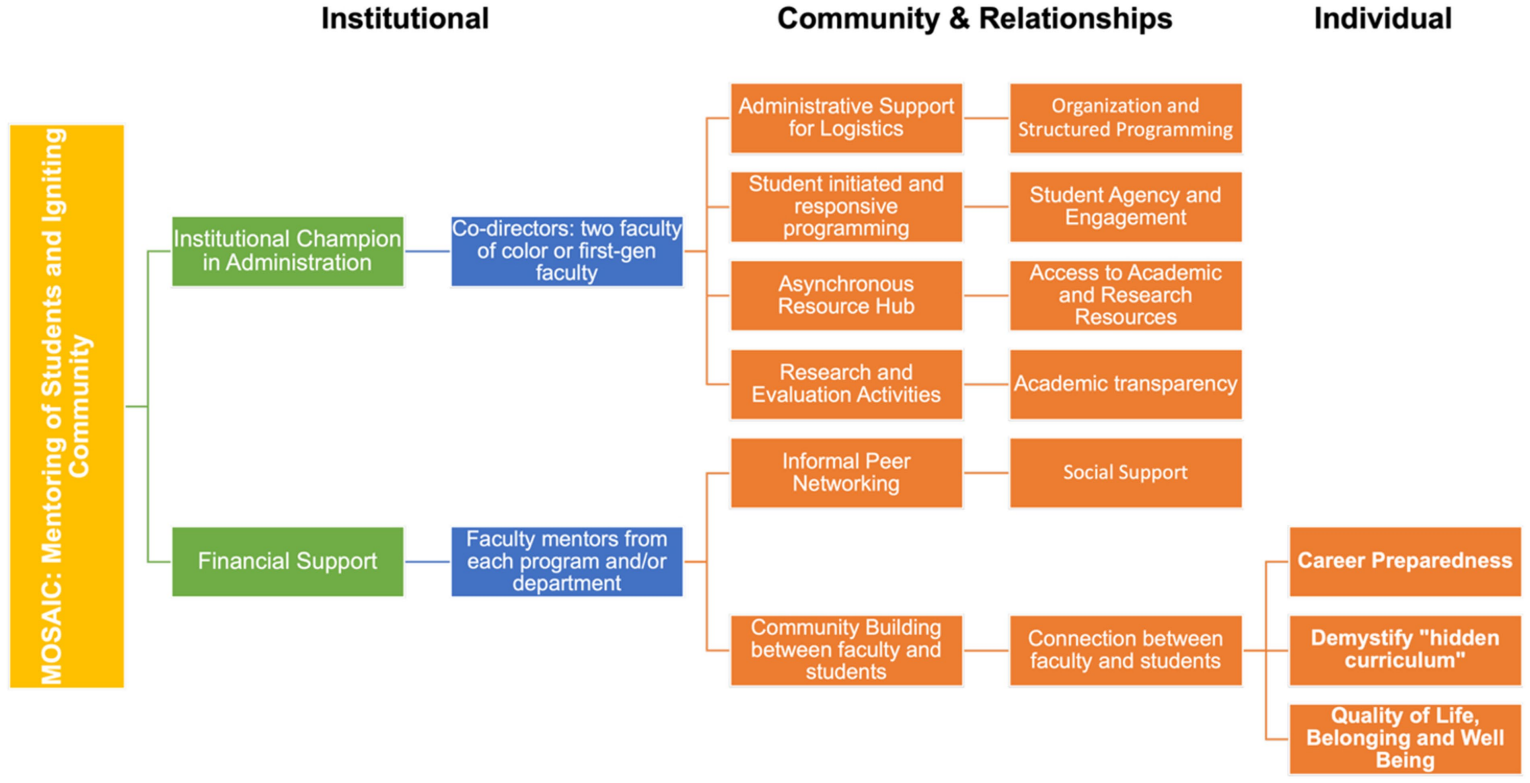
MOSAIC program model.

The former co-director of MOSAIC at Columbia, in partnership with a new co-director at USC, launched MOSAIC at USC in the Department of Population and Public Health Sciences (DPPHS) for doctoral students in August 2024. A key component of the MOSAIC adaptation was the incorporation of Culturally Responsive and Racially Equitable Evaluation and Engagement Strategies (CRREEE), relying on student input and fostering meaningful mentorship relationships that prioritize racial equity. The MOSAIC USC co-directors adapted and implemented the institutional and community inputs from the MOSAIC programmatic framework (Figure 2). Departmental leadership at USC expressed interest in supporting efforts aimed at broadening the reach of and addressing gaps in existing institutional equity initiatives.

Despite organizational willingness, challenges emerged in securing implementation funding due to institutional budgetary constraints, and limited capacity to support new initiatives. Ongoing deliberations were held in partnership with identified institutional advocates, specifically the Chair of DPPHS and the Vice Chair of Diversity, Equity, and Inclusion. These discussions emphasized the necessity of embedding key resource needs into the initially proposed budget. At any scale, this included funding for administrative support, stipends for faculty division leaders and other collaborators, as well as allocated activity and resource funding. MOSAIC at USC ultimately secured financial resources from contributions from the Dean’s Office at the USC Keck School of Medicine and existing allocations for the USC DPPHS REDI Council. That ensured that resources were available for MOSAIC for the academic year. During its first year, MOSAIC at USC received funding that allowed for, at minimum, the maintenance of program integrity and operational feasibility. Insights gained from the established MOSAIC program at Columbia MSPH pointed to the importance of strategic funding requests that ensured a sustained department-wide or school-wide budget past the first year, avoiding gaps that may cause implementation disruptions and minimizing the barriers in securing or relying on unreliable external funding streams.

MOSAIC was positioned as a key dimension of student-facing programming within the Department’s REDI Council. The initial planning and early implementation of MOSAIC at USC aligned with institutional priority areas designed to enhance professional development and success across its diverse cohorts of doctoral students. MOSAIC had institutional champions in DPPHS leadership and financial support. It was also established that MOSAIC would serve doctoral students exclusively until there were additional resources and support to expand the program to serve master’s students. MOSAIC was to be co-directed by two faculty members of color or first-generation faculty. MOSAIC also included a faculty mentor from each of DPPHS doctoral programs (three faculty members who identify as BIPOC, first-gen, or international). In total, five faculty members are involved and available for MOSAIC students at USC. All faculty involved in the implementation of the MOSAIC program were compensated for their efforts. This is an important aspect to alleviate excessive unpaid requests from the few BIPOC and first-generation faculty members in the department who met the criteria for MOASIC mentors, a shared identity with students and a demonstrated commitment to supporting underrepresented students. Often, BIPOC, first-generation, international faculty are asked to mentor students from similar backgrounds, helping them navigate a predominantly White environment. MOSAIC faculty mentors and directors must be compensated for their time in MOSAIC as a signal of administrative commitment for anti-racist structural change. All involved faculty are expected to provide group mentorship and participate in MOSAIC events. Faculty directors are to coordinate all aspects of MOSAIC, including program planning and evaluation, and faculty mentors are to be additional tethers and mentors for doctoral students in their specific doctoral programs.

To address the community and relationships dimensions of the programmatic framework (Figure 2), the MOSAIC team included administrative support for logistics, planning of events, listserv coordination, and scheduling. MOSAIC at USC includes asynchronous resources that are shared via a listserv. Research and evaluation were planned from the onset to verify program effectiveness and opportunities for program improvement. Given the smaller cohort of doctoral student participants in MOSAIC at USC in its initial year, evaluation activities to date have focused on process related outcomes, evaluation of events, and feedback on MOSAIC programming. For example, following a workshop series on writing for publications, students completed questionnaires about the workshop series to help shape future MOSAIC events (Table 2). Research and evaluation activities also enable MOSAIC students to engage in the research process (e.g., creating data collection tools, contributing to manuscript writing related to MOSAIC, etc.)

**Table 2:** MOSAIC events for doctoral students in year 1 (2024–25) at USC.

Event	Description	Semester
Program Introduction	An orientation event for doctoral students to introduce them to the MOSAIC program. Students can meet peers and faculty mentors and provide feedback to help shape future programming.	Fall
Reflection on the Semester	An informal event where students gather to discuss their experiences throughout the semester. This event promotes a sense of community, allowing MOSAIC students to share insights, address challenges, and build connections with peers.	Fall
Update on Department Changes	A session that provided MOSAIC doctoral students with updates and changes that were occurring in the department, giving them the opportunity to ask questions and provide suggestions for doctoral student engagement within the Department.	Fall
Chat with the Chair	A session providing MOSAIC students with the opportunity to engage in open dialogue with the Department Chair.	Spring
Listening Session and Lunch	A facilitated discussion creating a space for solidarity, dialogue, and collective action in response to the current political climate.	Spring
Writing for Publication: Editors Perspectives and Fundamentals, Workshop Part 1	The first session in a workshop series designed to guide doctoral students through the publication process in Public Health. This session focuses on the editorial perspective and the fundamentals of manuscript preparation, equipping students with the foundational skills needed for successful publication.	Spring
Writing for Publication: Reading for Research, Workshop Part 2	The second session in a three-part workshop series designed to develop students’ critical reading skills. MOSAIC students learn strategies for analyzing dense academic texts and gain practical techniques for synthesizing research literature.	Spring
Writing for Publication: Components of a Manuscript and Submission, Workshop Part 3	The third session in a three-part workshop series focuses on refining manuscript submissions for public health journals. This session covers strategies for writing and revising critical sections to strengthen the quality and impact of students’ submissions.	Spring
Summer Gathering and Lunch	An informal gathering designed to check in and foster community and strengthen connections among doctoral students participating in MOSAIC. Participants enjoy a casual meal while engaging in peer discussions on academic and professional topics, providing an opportunity to build relationships.	Summer

Leveraging the CRREEE approach, for the first MOSAIC session, all doctoral students were invited to participate and self-select into the program. Students of color and first-generation students self-select into MOSAIC, and they can participate as much or as little as they like. Importantly, there are no requirements. Doctoral students were notified about MOSAIC through student facing listservs as well as flyers posted in the relevant buildings. During the first meeting, DPPHS doctoral students were asked to brainstorm areas where they needed additional support and would like to see programming. This aligns with CRREEE and the MOSAIC programmatic tenet of student-initiated and aligned programming. Over the course of the year, the MOSAIC programming reflected the doctoral students requests from the first session. Table 2 shows the programming that was completed for MOSAIC over the course of a year (9 events in total, about one per month of the academic year). For example, doctoral students expressed a need for additional support with academic writing and publishing, and MOSAIC programming included a three-part “Writing for Publication” series. Conducting these workshops as a MOSAIC community, including MOSAIC faculty mentors and peers, creates a brave space to ask questions and discuss shared experiences, effectively reducing barriers to learning, such as anxiety and embarrassment, for a skill many graduate students are expected to master. Throughout the year, MOSAIC faculty and students shared space and fostered a sense of community. There were spillover effects in terms of peer-to-peer mentoring as the students’ shared tips and lessons learned with one another during MOSAIC events.

Since its implementation, MOSAIC at USC has engaged 15 to 20 doctoral students approximately once or twice a month (17–23% of BIPOC doctoral students in PPHS). In 1 year, faculty engagement in mentorship and anti-racist pedagogy has been significantly strengthened across the department, fostering a culture of inclusivity and academic support. There are three separate doctoral programs within the Department of Population and Public Health Sciences, and MOSAIC has helped bring these three programs, their students, and the faculty together for MOSAIC and the academic community. Students often comment that MOSAIC events are the only place where they have interactions with faculty and students from other programs within the department. Institutional commitment to equitable mentorship structures has also been enhanced, leading to greater integration of MOSAIC’s principles within the support offered to public health students across the department. Additionally, the program has served as a replicable model for faculty-to-student mentorship, demonstrating its potential for broader application across public health institutions. The pilot cohort at USC confirmed the model’s transferability and underscored the ongoing need for structured, anti-racist mentorship programs as departments strive to build equitable environments.

## Discussion

The development of the MOSAIC Program Model and implementation and adaptation at USC PPHS highlight the importance of institutional support, the value of student-driven program design, and the power of community-building as a mentorship approach for faculty and students. The program’s successful adaptation at USC underscores the potential for faculty-to-student group mentorship to serve as a catalyst for broader institutional transformation. As academic public health institutions strive to achieve inclusive excellence, structured mentorship programs like MOSAIC play a crucial role in dismantling systemic barriers.

### Adapting MOSAIC at other schools and programs of public health

The MOSAIC framework is adaptable to diverse institutional settings and programs and schools of public health. Successful implementation requires a tailored approach, considering each institution’s culture, resources, and demographics (Table 3).

**Table 3:** Adapting the MOSAIC program at schools and programs of public health.

**1. Institutional self-assessment and commitment**
Address institutional positionality
Assess existing barriers and opportunities for BIPOC, international, and first-gen students
Secure institutional champion in department or school leadership
**2. Aligning priorities through participatory collaboration**
Involve and get commitment from BIPOC, first-generation, and/or international faculty and staff
Generate interest among BIPOC, international, and first-gen students
**3. Design equitable and tailored MOSAIC program**
Adapt core MOSAIC Programmatic Model with student and faculty input
Establish frequency of meetings, peer cohort activities, and professional development workshops
**4. Data collection and evaluation**
Establish a process evaluation plan for ongoing programmatic improvements
Choose meaningful outcomes with MOSAIC faculty and students
Lay foundation for long-term research on MOSAIC and established outcomes
**5. Cultivating an anti-oppression and anti-racist culture**
Ongoing dialogue with students and faculty about equity and addressing systems of oppression
Accountability for addressing discriminatory incidents
Create community and sense of belonging for BIPOC, international, and first-gen students and faculty

Institutions should begin by assessing existing barriers and opportunities for BIPOC, international, and/or first-generation student success. This includes an understanding of institutional positionality as well as evaluating current mentorship structures, faculty diversity, and institutional policies (2, 16). Strong, visible commitment from institutional leadership to anti-racism and equity initiatives is essential, translating into dedicated funding, staffing, and integration of equity goals into strategic plans. Institutions that may already have the infrastructure and active programming aimed at inclusive student programming may be more interested in program expansion efforts. In contrast, those institutions that are less resourced and have limited administrative capacity for equity initiatives may focus on operationalization and institution-wide policy changes. Regardless of a restrictive funding environment or limited institutional support, MOSAIC can still be implemented through collaborative efforts with other existing initiatives that parallel its goals and objectives (34). Institutional buy-in, anchored by intentional leadership commitment and advocacy, facilitates resource and funding allocation, ensures inclusion in strategic plans, and lays the foundation for long-term sustainability (35). Institutionalization of MOSAIC programming in this way is critical to its long-term sustainability within unpredictable funding environments.

The next step of adaptation for MOSAIC requires an alignment of priorities through participatory collaboration and buy-in from relevant stakeholders. Adapting MOSAIC requires broad engagement across the institution, involving faculty, staff, administrators, current students, and alumni in planning and implementation (21). Engaging BIPOC, international, and/or first-generation faculty as MOSAIC mentors is ideal, given that many will have personal experience with navigating PWIs. Additional training on inclusive mentorship and implicit bias may be necessary for faculty and staff who will serve as mentors or program facilitators (36–38). Incentivizing participation through professional development credits or recognition can foster greater buy-in (38). Institutions should prioritize cultivating a diverse pool of faculty and staff mentors, particularly those who identify as BIPOC, international, and/or first-generation, to facilitate the community group mentorship model and distribute the mentorship load more equitably (5, 39).

Then, there should be a focus on tailoring the MOSAIC program to meet institutional and participant needs. While MOSAIC’s core principles (student driven programming, asynchronous resources, informal peer networking, and community-building between faculty and students) remain consistent, specific activities and delivery methods should be customized. The frequency and format of group mentorship meetings, peer cohort activities, and professional development workshops can be adjusted to align with institutional capacity and student needs. Institutions should prioritize engaging and compensating BIPOC, international, and/or first-generation faculty to conduct workshops or trainings (40, 41). When faculty who do not share these identities are not present, consider integrating MOSAIC workshops with existing resources (e.g., writing centers, counseling services) to leverage and amplify them. Emphasis should be placed on creating group-based mentorship opportunities and fostering peer-to-peer connections to build a strong sense of community, rather than solely relying on individual pairings (21, 22).

To ensure sustained impact and continuous improvement, adapting institutions should aim to include robust data collection and evaluation efforts for MOSAIC. The process evaluation plan can help inform programming and whether student needs are being met. In the long term, meaningful outcomes should be selected with input from students and faculty. These may include things like student retention and completion rates, assessing student perceptions of inclusivity and support through surveys, and gathering feedback from mentors and mentees following workshops and training activities. Consider engaging institutional leadership to incorporate questions about student engagement and experience with MOSAIC as part of their exit and alumni surveys. Finally, investment in longitudinal studies, while costly, can help ascertain long-term impacts on career trajectories and leadership roles. This data is vital for demonstrating program effectiveness and for ongoing institutional support.

Lastly, beyond programmatic elements, successful adaptation of MOSAIC hinges on fostering an institutional culture that actively engages in anti-oppressive pedagogy and aims to dismantle racism to create a sense of belonging for BIPOC, first-generation, and international students in higher education (4, 42, 43). This involves ongoing dialogues about equity, accountability for addressing discriminatory incidents, and creating a sense of community and belonging for students and faculty (42). By embedding anti-oppression principles into the institution’s fabric, the MOSAIC framework can thrive and contribute to a more equitable academic landscape for all students.

Given the potential expansion of MOSAIC across diverse environments, the MOSAIC Program Model (Figure 2) holds support elements alongside key features to allow for modification, assuring the program elements remain standardized and flexible. Tracking fidelity according to MOSAIC’s key features involves tangible institutional support, a faculty to student group mentorship approach to build community, and an established feedback loop regarding programming among program participants. MOSAIC’s framework allows for and encourages flexibility in its program structure, recognizing that it is shaped by the unique culture and organizational structure of each institution, the distinct needs of their student body, and what is relevant and what is reasonable within those contexts.

The value in a program like MOSAIC is its replicability across various institutional setting due to its iterative and responsive model. The success of MOSAIC across institutions lies in its ability to remain adaptive. The adaptation of MOSAIC for DPPHS doctoral students at USC demonstrates how group mentorship can be leveraged as an adaptive change strategy, fostering a more equitable and supportive academic environment in any program or school of public health. By sharing our experiences, we hope to provide the MOSAIC Programmatic Model for other institutions seeking to transform public health education and cultivate a diverse, competent, and justice-oriented workforce.

## Data Availability

The original contributions presented in the study are included in the article/supplementary material, further inquiries can be directed to the corresponding author.
